# Integrating vaginal laser therapy into multidisciplinary management of bladder pain syndrome and vulvodynia

**DOI:** 10.1186/s12894-026-02114-4

**Published:** 2026-03-27

**Authors:** Nobuo Okui

**Affiliations:** 1 Innovative Longevity, Juntendo University, Tokyo, 113-8421 Japan; 2 Data Science, Kanagawa Dental University, Inaoka 82, Yokosuka, Kanagawa 238-008 Japan; 3 Urogynecology, Yokosuka Urogynecology and Urology Clinic, Ootaki 2-6, Yokosuka, Kanagawa 238-0008 Japan

**Keywords:** Laser therapy, Bladder pain syndrome, Interstitial cystitis, Vulvodynia, Er:YAG, Nd:YAG, Transvaginal photobiomodulation

## Abstract

**Background:**

Bladder pain syndrome/interstitial cystitis (BPS/IC) and vulvodynia frequently co-occur, forming overlapping pelvic pain syndromes that often evade organ-centered management. Shared mechanisms—urothelial/epithelial dysfunction, neuroinflammation, and pelvic floor hypertonicity—support multidisciplinary, mechanism-based care. Because high-quality clinical evidence specifically targeting the BPS/IC + vulvodynia overlap remains limited, this narrative review also evaluates laser-based therapies in clinically adjacent conditions with shared anatomy and pathophysiology (e.g., genitourinary syndrome of menopause and vulvar dermatoses) to contextualize plausibility and inform future study design.

**Main body:**

Non-ablative erbium-doped yttrium aluminum garnet (Er:YAG) and neodymium-doped yttrium aluminum garnet (Nd:YAG) vaginal/vulvar protocols have shown encouraging improvements in superficial vulvar pain, dyspareunia, sexual function, and—in selecte cohorts—bladder-related symptoms among patients with coexisting BPS/IC and vulvodynia. Transvaginal photobiomodulation (TV-PBM) is emerging as another non-ablative modality with putative mitochondrial, anti-inflammatory, and neuromodulatory effects. Across modalities, histological and imaging reports describe epithelial thickening, glycogen restoration, neovascularization, and collagen remodeling, consistent with tissue repair within the urogynecologic–pelvic floor unit. Proposed mechanisms include sublethal photothermal activation of heat-shock responses, modulation of microvascular tone, and attenuation of inflammatory mediators. Nevertheless, current evidence is dominated by small case series and non-controlled studies with heterogeneous parameters and short follow-up; long-term efficacy, safety, dose, targets, and schedules remain to be standardized. Recent clustering and phenotyping work highlights BPS/IC subtypes—including vulvodynia-predominant groups with distinct psychological and quality-of-life profiles—suggesting that responses to laser modalities are likely phenotype-dependent. In parallel, natural-language analyses reveal “semantic drift” between clinical terms and patients’ everyday symptom language, indicating a role for AI-assisted processing of diaries and free-text to refine phenotyping and endpoint selection.

**Conclusions:**

Vaginal/vulvar laser therapy and TV-PBM can be positioned as mechanism-specific modules within individualized, multidisciplinary care for BPS/IC and vulvodynia. Priority next steps include adaptive or stratified randomized trials, harmonized energy/targeting protocols, multidimensional patient-reported outcomes, and rational combinations with pelvic floor rehabilitation, hormonal or androgen-sparing approaches, and psychological interventions. Incorporating AI-based profiling and vocabulary mapping into clinical workflows may sharpen subtype recognition and treatment targeting, ultimately improving outcomes for patients with overlapping BPS/IC and vulvodynia.

## Background

Bladder Pain Syndrome/Interstitial Cystitis (BPS/IC) and vulvodynia are chronic, debilitating conditions characterized by persistent pain localized to the bladder and vulvar regions, respectively. These disorders are particularly prevalent among women of reproductive age and are known to substantially impair daily functioning and overall quality of life (QoL) [1, 2]. Conventional management approaches—including pharmacological therapies, behavioral interventions, and pelvic floor physical therapy—frequently result in limited or short-term symptom relief.

BPS/IC is defined by ongoing pelvic pain or pressure perceived to originate from the bladder, often accompanied by urinary urgency and increased frequency [3]. Despite considerable research efforts, the precise pathophysiological mechanisms remain incompletely understood. Current evidence points toward a multifactorial etiology involving urothelial dysfunction, dysregulated immune responses, and neurogenic inflammation, all of which may contribute to the persistence and complexity of symptoms [4].

In recognition of this clinical complexity, a recent international consensus has identified five major gynecologic comorbidities that frequently coexist with BPS/IC: (1) endometriosis/adenomyosis, (2) genito-pelvic pain/penetration disorder, (3) overactive pelvic floor muscles, (4) hormone-associated genitourinary changes, and (5) vulvodynia/vestibulodynia [1]. These conditions often overlap symptomatically with BPS/IC and may influence both clinical presentation and therapeutic responsiveness. Therefore, a comprehensive assessment that integrates gynecologic findings is now increasingly emphasized in BPS/IC care.

In recent years, laser-based therapies have emerged as a promising minimally invasive modality capable of targeting both epithelial and neuromuscular components of pelvic pain syndromes [5–9]. Furthermore, this review considers laser-based treatments for related gynecologic conditions that share symptomatic or mechanistic overlap with BPS/IC—such as vulvar lichen sclerosus and genitourinary syndrome of menopause (GSM) observed in specific populations (e.g., breast cancer survivors) [9–13].

**Fig. 1:**
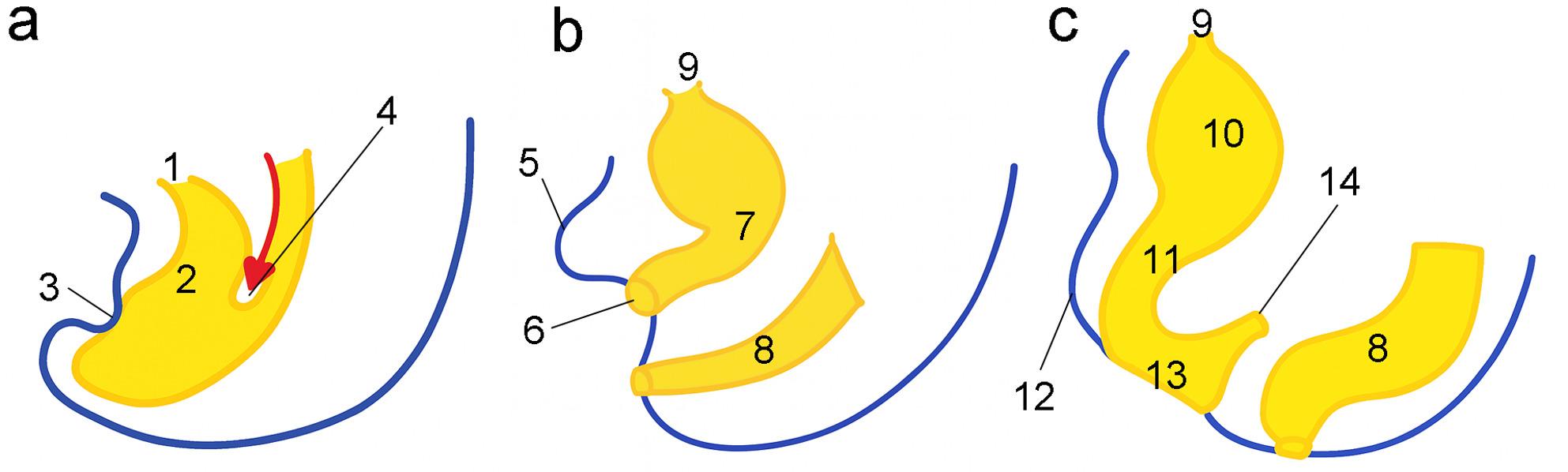
Development of the female urogenital system. **a** Week 7:1: Allantois 2. Cloaca 3. Cloacal membrane 4. Urorectal septum **b** Weeks 8–9:5. Genital tubercle 6. Perforation of urogenital membrane 7. Urogenital sinus 8. Rectum 9. Urachus **c** Week 12:9. Urachus 10. Urinary bladder 11. Urethra 12. Clitoris 13. Vestibule 14. Lower portion of rectum

This narrative review summarizes the current body of evidence regarding the use of laser treatment in BPS/IC and vulvodynia, with a focus on therapeutic efficacy, safety profiles, and potential mechanisms of action. Special attention is given to differential treatment responses among patient subgroups, the biological plausibility of tissue remodeling effects, and the pressing need for standardized protocols to guide clinical application. To contextualize these clinical phenomena, Fig. 1 illustrates the embryological development of the female urogenital system across three critical stages. Stage A (Week 7) depicts the early cloacal configuration, characterized by a common urogenital and anorectal chamber, with the urorectal septum descending toward the cloacal membrane. Stage B (Weeks 8–9) shows the perforation of the urogenital membrane and the initial separation of the urogenital sinus from the rectum, alongside the emergence of the genital tubercle. Finally, Stage C (Week 12) represents the completion of endodermal differentiation, with distinct anatomical structures such as the bladder, urethra, vestibule, and clitoris now established. This developmental progression illustrates the shared embryological origin of the lower urinary and genital tracts, offering an anatomical explanation for the clinical overlap observed in BPS/IC and vulvodynia [14].

## Main Text

### Vulvodynia and its overlap with BPS/IC: prevalence, pathophysiology, and clinical impact

Vulvodynia is defined as persistent vulvar discomfort or pain without a clear identifiable cause, typically lasting for at least three months [15]. Patients often describe symptoms such as burning, stinging, or irritation, which may be spontaneous or triggered by physical contact [15, 16]. The underlying mechanisms are considered multifactorial, involving peripheral nerve sensitization, inflammatory responses, hormonal influences, and genetic predispositions [15–17]. These complex and overlapping factors frequently complicate diagnosis and long-term management.

Epidemiological studies have shown that BPS/IC affects approximately 2.7% to 6.5% of women [3, 4, 17], while vulvodynia occurs in around 8% of women by the age of 40 and up to 16% over a lifetime [18, 19]. Both conditions are associated with significant reductions in QoL, including impaired sexual function and significant psychological distress [20].

Gardella et al. reported that among women newly diagnosed with IC, 23.4% experienced spontaneous vulvodynia, and 74.5% reported provoked vulvodynia [21]. These individuals also exhibited significant sexual dysfunction, with a mean Female Sexual Function Index (FSFI) score of 16.85 ± 8.73, significantly lower than that of healthy controls (27.34 ± 6.41; *p* < 0.0001). Similarly, the UNICORN-1 study by Okui et al. found that Asian women with BPS/IC had markedly lower FSFI scores (15.72 ± 4.46 vs. 26.3 ± 4.93; *p* < 0.05) and higher vulvodynia swab test scores than controls [22].

In a study of 1,183 women with chronic vulvar pain, 64.2% identified superficial dyspareunia as their primary complaint, while 43.4% reported sexual desire or arousal disorders. Common comorbidities included recurrent vulvovaginal candidiasis (32%), urinary tract infections (37.4%), and irritable bowel syndrome (28%) [23]. Vestibulodynia was diagnosed in 70.8% of participants, while 27.3% met criteria for generalized vulvodynia.

Collectively, these findings underscore the intricate clinical and pathological links between BPS/IC and vulvodynia [24]. The overlap between the two conditions contributes to heightened sexual dysfunction and broader psychosocial burdens, emphasizing the need for integrated diagnostic and therapeutic approaches [9, 13–16]. Notably, a recent international consensus panel has formally recognized vulvodynia/vestibulodynia as one of the five major gynecologic comorbidities associated with BPS/IC, further highlighting the clinical relevance of this overlap [1].

### Cellular and neurological basis

Beyond this shared anatomical origin, bladder pain syndrome/interstitial cystitis and vulvodynia also overlap at the cellular and neuroinflammatory levels [25].

Mast cells are increasingly recognized as key mediators in chronic inflammatory disorders such as BPS/IC and vulvodynia. These immune cells secrete various proinflammatory molecules—including cytokines, neuropeptides, and vasoactive agents—that are implicated in the pathogenesis of stress-related neuroinflammatory conditions [26]. In BPS/IC, activated mast cells can enhance neural excitability and alter neurotransmitter dynamics, contributing to persistent pelvic pain and urinary dysfunction [27].

In vulvodynia as well, increased mast cell activation has been observed. Histamine released from these cells is thought to activate peripheral nociceptors such as C-fibers and A-delta fibers, enhancing nociceptive signaling [28, 29]. Women with vulvodynia have been shown to exhibit increased pressure pain sensitivity not only in the vulvar area but also at distant body sites, suggesting a role for central sensitization in symptom generation [28]. Furthermore, patients with provoked vestibulodynia demonstrate impaired diffuse noxious inhibitory control (DNIC), indicating dysfunction in endogenous pain inhibition. These individuals also tend to show globally lowered pain thresholds compared to healthy controls, reflecting a generalized hypersensitivity state [29].

A recent cross-species study has further advanced our understanding of mast cells by identifying distinct subsets with organ-specific transcriptomic profiles in both mice and humans [30].

### Neurogenic inflammation, neuropathic pain, and central sensitization

The involvement of both peripheral and central nervous systems in the pathophysiology of BPS/IC and vulvodynia is intricate and not yet fully elucidated. Clinical findings suggest the presence of subtypes, with pain arising either from peripheral organs or generated centrally within the nervous system pathways [25, 31]. The female urinary and reproductive systems share innervation through the sacral nerves, encompassing both efferent (motor) and afferent (sensory) branches of the somatic and autonomic systems [27, 32]. Neurons in the dorsal root ganglia at the thoracolumbar and sacral levels are critical for relaying nociceptive signals from the pelvic region to the brain [28, 33]. In individuals with BPS/IC, alterations have been observed in endogenous descending pain inhibition mechanisms, such as those typically assessed by conditioned pain modulation [28, 33]. Pain experienced by patients with BPS/IC and vulvodynia often is often neuropathic in nature. This type of pain results from nerve damage or hypersensitization, causing neurons to fire spontaneously or in response to normally non-painful stimuli, leading to allodynia [28, 33, 34]. In particular, women with provoked vestibulodynia show amplified sensory responses in the genital area, a pattern also observed in other hypersensitivity disorders [34]. Evidence of abnormal central pain processing, indicative of central sensitization, has been reported in both BPS/IC and vulvodynia. A recent functional magnetic resonance imaging (fMRI) study by Hampson et al. assessed brain responses to pressure stimuli applied to both the vulva and a remote site (thumb) in women with vulvodynia (*n* = 24), healthy controls (*n* = 13), and fibromyalgia patients (*n* = 24). Women with vulvodynia and fibromyalgia exhibited significantly greater activation in the insular cortex in response to thumb pressure compared to healthy controls (*p* < 0.005) [35]. In patients with BPS/IC, fMRI studies have similarly shown increased functional connectivity in sensory and motor networks relative to controls. This enhancement was most prominent in individuals who reported pain during bladder filling [31]. Furthermore, a recent review highlighted central sensitization in both vulvodynia and endometriosis, noting associated findings such as altered serum biomarkers, differences in resting-state brain networks, and lowered pain thresholds [36].

### Hormonal influences

Emerging research has emphasized the influence of hormonal factors on central pain sensitization and nociceptive signaling. Estrogen, in particular, plays a modulatory role in pain perception mechanisms within the nervous system [37], and consistent sex-based differences in pain sensitivity and responses to analgesics have been reported [38]. Local estrogen application has been explored as a therapeutic option for both BPS/IC and vulvodynia [39], while the broader influence of gonadal hormones on sex-specific pain modulation and opioid efficacy has also been underscored [38]. Disruption of endocrine function may contribute to symptom onset and persistence in these disorders. For instance, premenstrual increases in estrogen may aggravate symptoms by stimulating mast cell degranulation and enhancing substance P release [26, 40, 41]. Gonadal hormones are also known to affect pain-related neuromodulatory systems in the central nervous system [37, 42]. Some studies have identified a link between the use of combined oral contraceptives and increased vulvodynia risk [43–45], though not all investigations have confirmed this association [45, 46]. Further research is warranted to clarify how both endogenous hormone levels and exogenous hormone therapies influence the etiology and progression of BPS/IC and vulvodynia. More recently, serum testosterone levels have garnered attention in patients presenting with lower urinary tract symptoms. While earlier reports suggested that testosterone deficiency was associated primarily with stress urinary incontinence, newer data indicate a correlation between low testosterone and symptoms such as urgency and overactive bladder, particularly in frail individuals—raising the possibility of testosterone-based treatment strategies for severe BPS/IC cases [47, 48]. In postmenopausal women, vulvodynia has been linked to reduced levels of estradiol, testosterone, and dehydroepiandrosterone sulfate [48]. Given testosterone’s role in preserving genital tissue health and sexual function, non-estrogen therapies—such as vaginal testosterone, dehydroepiandrosterone, or laser treatments—are being utilized to manage the genitourinary syndrome of menopause [49].

Beyond these core disorders, hormonal modulation also plays a crucial role in related urogenital conditions, such as genitourinary syndrome of menopause and vulvar lichen sclerosus [10, 11, 50]. Although vulvar lichen sclerosus is a distinct pathological condition from idiopathic vulvodynia, it is notable for sharing anatomical proximity and symptom overlap as a chronic vulvar disorder [51]. In a prospective study of postmenopausal women with lichen sclerosus, short-term topical testosterone treatment (0.04 g daily for 4 weeks) significantly improved pain scores and epithelial atrophy, although some degree of systemic absorption and mild increases in serum testosterone were observed in several patients [10]. However, subsequent systematic reviews and international guidelines have not supported the routine use of topical androgens for lichen sclerosus, citing insufficient evidence of efficacy compared with ultrapotent corticosteroids [52–54]. The 2018 British Association of Dermatologists guidelines make no recommendation regarding testosterone, and both the Cochrane review and the EMAS clinical guide conclude that high-potency topical corticosteroids remain the first-line therapy, with no additional benefit demonstrated for estrogen or testosterone creams [53, 54]. These data are mentioned here only as contextual evidence from related vulvar dermatoses, to contrast the limited hormonal findings in BPS/IC and vulvodynia.

### Diagnosis and comorbid conditions associated with vulvodynia

The clinical diagnosis of vulvodynia is primarily based on a detailed medical history and a comprehensive pelvic examination. During the examination, the cotton swab test is commonly employed to evaluate vulvar sensitivity. One cross-sectional study in postmenopausal breast cancer survivors with moderate-to-severe GSM-related dyspareunia systematically applied this test to multiple vestibular and periurethral sites, including the hymenal remnants, to map the exact location of pain and explore its potential causes [12]. The investigators found that almost all women localized their maximal pain to the vulvar vestibule just outside the hymen, whereas provoked pain in the vaginal walls was uncommon, challenging the traditional view that postmenopausal dyspareunia is primarily driven by intravaginal dryness [12]. While the cotton swab test is considered a reliable tool for monitoring treatment responses in vulvodynia [55], it is noteworthy that some affected individuals may not demonstrate heightened vulvar sensitivity [56]. In rare cases, such as a pediatric patient with Mayer-Rokitansky-Küster-Hauser syndrome, vulvodynia and symptoms of overactive bladder may present atypically, potentially leading to misdiagnosis as a neurodevelopmental disorder [57].

Research examining women diagnosed with both BPS/IC and vulvodynia indicates frequent comorbidity and a significantly increased prevalence of depressive symptoms in those with vulvodynia. Moreover, histories of psychological or sexual trauma have been linked to co-occurring pelvic floor hypertonicity, vulvodynia, and major depressive disorder [58].

**Fig. 2:**
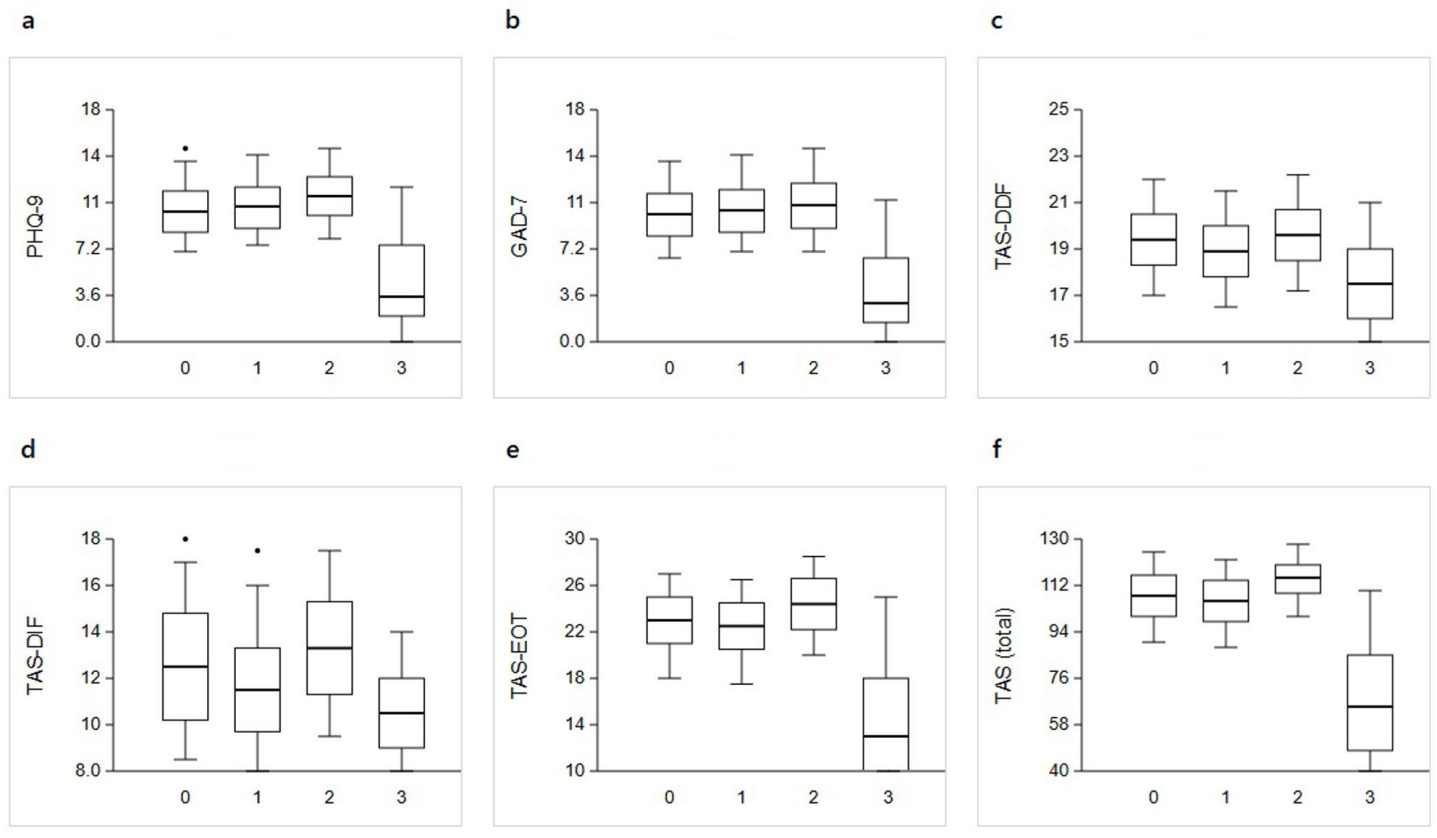
Psychological and emotional characteristics by cluster. Boxplots of six psychological assessment scales: **a** PHQ-9 (depressive symptoms), **b** GAD-7 (anxiety), **c** TAS-DDF (difficulty describing feelings), **d** TAS-DIF (difficulty identifying feelings), **e** TAS-EOT (externally oriented thinking), **f** TAS total score. X-axis: cluster number (0, 1, 2, 3); Y-axis: score on each respective scale.Clusters 0, 1, and 2: BPS/IC patient subgroups; Cluster 3: control group. Highest scores across all measures in Cluster 2;lowest scores in Cluster 3.Cluster 2: vulvodynia-predominant, non-urologic phenotype with elevated psychological distress and alexithymia-related traits

A cross-sectional study of 150 women with comorbid BPS/IC and vulvodynia identified a vulvodynia-predominant subtype characterized by severe sexual dysfunction and psychological distress [59]. Figure 2 shows the distribution of depressive symptoms, anxiety, and alexithymia-related traits across the three BPS/IC clusters and the control group identified by the UNICORN-4 analysis, which used validated instruments for psychological distress and alexithymia (PHQ-9, GAD-7, TAS-20) [59]. The vulvodynia-predominant cluster showed the highest vulvar pain scores on the swab test, relatively mild bladder symptom scores, and significantly greater depressive and anxiety symptoms than the other clusters and controls, together with more pronounced alexithymic traits. However, some aspects of sexual function and distress in women with BPS/IC and vulvodynia may not be fully captured by standard instruments such as the Female Sexual Function Index (FSFI) and the Female Sexual Distress Scale–Revised (FSDS-R) [59]. These findings support the concept of a non-urologic pelvic pain phenotype in a subset of BPS/IC patients and underscore the need to integrate psychological profiling and more tailored sexual function measures into the assessment of BPS/IC–vulvodynia comorbidity.

Similar psychological profiling approaches have also been applied in other nociplastic pain conditions such as fibromyalgia and chronic headache, where multidimensional assessments of traumatic experiences, defense mechanisms, and alexithymia have been used to characterize distinct psychological profiles across chronic pain syndromes [60].

### Vaginal laser modalities for BPS/IC and vulvodynia

Laser therapy targeting the pelvic pain complex of BPS/IC and vulvodynia has primarily focused on non-ablative Erbium-doped yttrium aluminum garnet (Er:YAG) and Neodymium-doped yttrium aluminum garnet (Nd:YAG) laser systems [6–8, 57, 61–63], and transvaginal photobiomodulation (TV-PBM) [64]. These modalities share regenerative and neuromodulatory mechanisms within the vaginal and periurethral tissues, aiming to alleviate chronic pelvic pain and restore mucosal function. The Er: YAG laser (2,940 nm) demonstrates high absorption by water with minimal tissue penetration, enabling precise and superficial photothermal modulation [65–67]. Its effects appear to operate within a variable heat-shock response model, replacing the classical Arrhenius principle of exponential thermal injury [65–68]. Sublethal heating activates heat-shock proteins and regenerative growth-factor pathways (such as VEGF and TGF-β), enhancing epithelial viability and angiogenesis (new vessel formation) under controlled thermal stress [66, 68]. This self-protective cascade promotes epithelial renewal, collagen remodeling, and neural modulation without irreversible injury [66–68]. Such a mechanism provides a biological rationale for the durable improvements observed in both BPS/IC and vulvodynia [6–8], as intense Heat-Shock Biomodulation (i-HBM) is known to alleviate related “urinary symptoms such as frequency, urgency and dysuria” [68]. Clinically, limited pulse sequences (approximately 4–7 pulses over 1–3 s) induce superficial epithelial rejuvenation, while longer pulse trains (10–30 pulses over > 5 s) under local anesthesia reach subepithelial layers (400–500 μm), stimulating neocollagenesis (new pro-collagen type I) and microvascular repair without coagulative necrosis [62, 65, 66, 68].

The Nd: YAG laser (1,064 nm) penetrates more deeply due to its lower water absorption, producing submucosal photothermal effects on vasculature and connective tissue [57, 67, 69]. When used in combination, Er: YAG and Nd: YAG dual-wavelength regimens can synergistically restore mucosal elasticity, enhance vascular perfusion, and modulate nociceptive pathways—yielding measurable improvements in pain, urgency, and dyspareunia among BPS/IC and vulvodynia patients [7, 8, 69].

TV-PBM represents another non-ablative strategy aimed at bladder modulation and pelvic pain reduction [64]. Near-infrared light stimulates cytochrome c oxidase in mitochondria, increasing oxygenation, suppressing inflammatory cytokines, and regulating nitric oxide and reactive oxygen species [64]. At the cellular level, PBM augments ATP synthesis, reduces oxidative stress, and improves smooth muscle relaxation—leading to analgesia and improved bladder function [64].

Collectively, these findings establish TV-PBM as a core-domain therapeutic option, analogous to Er: YAG and Nd: YAG lasers, within the multimodal management of BPS/IC and vulvodynia.

**Fig. 3:**
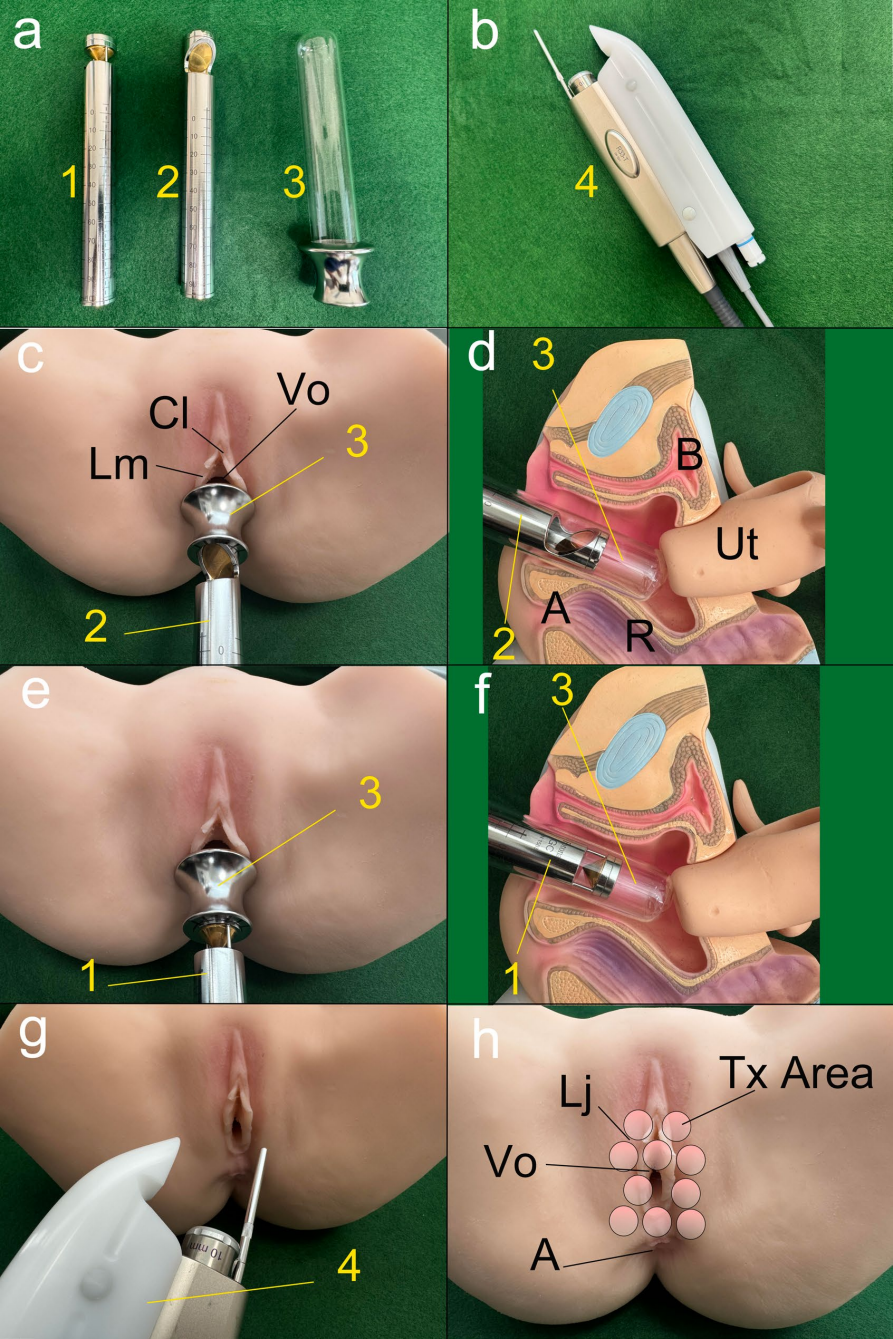
Step-by-step visualization of the laser devices, application techniques, and anatomical targets used in combined Er: YAG (VEL) and Nd: YAG laser therapy with the Fotona system. **a** Instruments used in the procedure.1: R11-GC1 handpiece for circumferential (360°) vaginal wall irradiation.2: PS03-GA handpiece for anterior vaginal wall irradiation.3: Glass speculum. **b**　4: R33 non-contact handpiece for external Nd:YAG laser irradiation (Fotona SP Dynamis, PIANO mode). **c** Frontal view showing irradiation of the anterior vaginal wall using the PS03-GA handpiece (2) inserted via the glass speculum (3).Anatomical landmarks include Cl (clitoris), Vo (vaginal opening), and Lm (labia minora). **d** Cross-sectional view corresponding to panel c, showing the PS03-GA handpiece (2) targeting the anterior vaginal wall.e: Frontal view showing circumferential irradiation of the vaginal wall using the R11-GC1 handpiece (1) inserted via the glass speculum (3) **f** Cross-sectional view corresponding to panel e, demonstrating 360° irradiation with the R11-GC1 handpiece (1) **g** Application of external Nd:YAG laser to the vulvoperineal region using the R33 non-contact handpiece (4) in PIANO mode (spot size: 9 mm; pulse: 5 seconds; fluence: 90 J/cm²) **h** Schematic representation of irradiation sites (Tx Area) including the labia majora (Lj), vaginal opening (Vo), and anus (A).AbbreviationsCl: Clitoris, U: Urethra, Vo: Vaginal Opening, Lm: Labia Minora, Lj: Labia Majora, Ut: Uterus, Cx: Cervix, V: Vagina, A: Anus, R: Rectum, Tx Area : Target Area

Figure 3 provides a visual overview of the laser instruments and anatomical targets involved in the application of Er: YAG and Nd: YAG therapy, including anterior and circumferential vaginal wall irradiation and external vulvoperineal treatment.

### Laser modalities for related gynecologic conditions

BPS/IC and vulvodynia straddle the interface of urology, gynecology, and pain medicine, and share vaginal and vulvar tissue as a common anatomic substrate; therefore, examining laser applications in adjacent gynecologic conditions can provide clinically relevant insights into mechanisms and translational potential for this comorbid pain complex. Beyond these core disorders, similar photothermal interventions have been applied to related gynecologic conditions with overlapping symptomatology and tissue targets, particularly the GSM [70]. The fractional microablative CO₂ laser (10,600 nm) has been widely investigated for GSM—a hypoestrogenic condition characterized by vulvovaginal atrophy, dryness, and dyspareunia—and is now one of the most extensively studied vaginal energy-based modalities [70–72]. Although direct clinical evidence for CO₂ laser therapy in BPS/IC is limited, randomized and observational data in GSM and breast cancer survivors suggest that fractional CO₂ can improve vaginal lubrication, epithelial elasticity, and superficial pain, thereby providing a potentially translatable model for overlapping pelvic pain syndromes [72, 73].

In fractional mode, CO₂ lasers generate controlled microthermal zones within the vaginal epithelium, initiating a wound-healing cascade that promotes epithelial regeneration, neovascularization, and collagen remodeling within the lamina propria [70, 71]. Histologic studies in postmenopausal atrophic mucosa demonstrate restoration of a thick, glycogen-rich stratified squamous epithelium, re-formation of connective-tissue papillae, and increased capillary density after fractional CO₂ exposure [70]. The absorption coefficient of CO₂ light in water is approximately one order of magnitude lower than that of Er: YAG, so delivered energy penetrates more deeply but with less spatial precision, favoring fractional over uniform superficial heating of the mucosa [70, 72].

These optical and tissue-interaction characteristics make CO₂ lasers particularly suitable for vulvar and vaginal pain associated with identifiable hypoestrogenic or inflammatory conditions, such as GSM with vestibular atrophy, rather than for idiopathic vulvodynia or BPS/IC itself [71–73]. Accordingly, CO₂-based interventions are best interpreted as adjacent-domain therapies—clinically informative for their shared regenerative and neuromodulatory mechanisms, but not as primary evidence for the management of BPS/IC [70–73].

### Histopathological effects of Er:YAG laser treatment

Significant tissue-level improvements have been observed following Er:YAG-based laser therapies in patients with BPS/IC and vulvodynia. In one reported case involving a postmenopausal woman, non-ablative treatment with Er:YAG and Nd:YAG lasers led to marked histological recovery. Initial bladder biopsies indicated pronounced inflammation, mucosal detachment, and notable immune cell infiltration. After treatment, regenerated urothelial tissue exhibited normalized mucosal thickness (increasing from 8.33 ± 12.1 μm to 106.7 ± 35.9 μm) and no presence of Hunner’s lesions. Vaginal samples also revealed beneficial changes, including epithelial thickening (from 57.3 ± 14.2 μm to 133.3 ± 24.5 μm), enhanced glycogen deposition, emergence of new papillae, and neovascularization within the lamina propria [8]. These morphological improvements corresponded with reductions in urinary frequency, dysuria, and vulvar pain, alongside improved vaginal health scores and bladder function parameters [8].

Additionally, a case series involving 10 women (mean age: 60.6 years) with advanced vaginal atrophy demonstrated notable histological regeneration after non-ablative Er:YAG laser application. Vaginal epithelial thickness increased markedly from a baseline average of 45 μm (range: 10–106 μm) to 153 μm (range: 97–244 μm) at three months post-treatment. The therapy facilitated expansion of epithelial layers (from 5 to 10 layers to 20–40), as well as enhanced glycogen accumulation, papillary architecture, collagen formation, and vascularization supporting the lamina propria and overlying epithelium [66].

### Clinical evidence for vulvodynia in BPS/IC patients

Multiple investigations have evaluated the clinical efficacy of laser-based therapies for BPS/IC, particularly in the context of vulvodynia comorbidity. The UNICORN-2 study demonstrated that repeated monthly vaginal Er:YAG laser (VEL) applications over one year yielded substantial symptom relief among patients with BPS/IC [6]. At 12 months, 75% of participants showed marked improvement, with average NRS-11 pain scores declining from 10.11 ± 0.92 to 2.09 ± 2.03, and further to 1.44 ± 1.33 by 18 months. ICSI scores improved from 15.6 ± 1.33 to 5.67 ± 2.17 and then to 4.56 ± 1.67, while ICPI scores decreased from 13.0 ± 1.50 to 4.78 ± 0.67 and 4.11 ± 0.33, respectively. Functional bladder capacity significantly increased from 68.78 ± 15.0 ml to 186.7 ± 25.0 ml, and urinary frequency was reduced from 18.5 ± 6.51 to 7.56 ± 1.58 times per day at 12 months. Notably, these improvements persisted for up to 18 months post-treatment, and no lasting adverse events were reported, although the precise mechanism remains undetermined [6].

Another investigation involving TV-PBM assessed its effectiveness in alleviating pelvic pain in women with BPS/IC through a multicenter observational cohort across 17 sites [64]. Among 140 participants, 89.3% completed at least four sessions and 59.3% completed all eight. The primary endpoint—a ≥ 2-point reduction in NRS-assessed pelvic pain after 8 sessions—was achieved by 63.9% of patients. Significant symptom relief was reported across various domains including urination, physical activity, and sexual function. Furthermore, the proportion of patients experiencing moderate-to-severe pain dropped from 83.1% to 38.5% (*p* < 0.001). Despite these positive outcomes, the underlying biological mechanism remains to be clarified.

The UNICORN-3 study examined the combined use of VEL and Nd:YAG lasers in women presenting with both vulvodynia and BPS/IC [7]. Fifteen patients underwent three treatment sessions, followed by evaluations at six and twelve months. The vulvodynia test scores declined from 9.73 ± 0.44 at baseline to 4.6 ± 0.48 at six months and 1.73 ± 0.68 at twelve months. Concurrently, NRS-11 bladder pain scores dropped from 9.53 ± 0.62 to 4.33 ± 0.47 and then to 1.33 ± 0.47. ICSI scores fell from 15.73 ± 1.77 to 8.53 ± 3.20 and 1.73 ± 0.77, and ICPI scores from 14.33 ± 1.07 to 4.87 ± 1.31 and 1.26 ± 0.92. PUF scores also improved substantially, decreasing from 21.47 ± 1.31 to 10.19 ± 1.47 and 2.8 ± 0.75. Mean voided volume rose from 54.13 ± 19.29 ml at baseline to 217.67 ± 54.49 ml at six months and 305.53 ± 40.93 ml at twelve months. Daily voiding frequency dropped from 24.6 ± 6.58 to 12.33 ± 2.91 and further to 6.33 ± 1.49. These findings support a synergistic short-term effect of dual laser modalities, with sustained long-term benefits potentially operating through distinct mechanisms.

Here is a rewritten version of the paragraph in English, retaining the original citation number [59] and avoiding potential plagiarism:

Earlier pilot work suggested that fractional CO₂ laser treatment applied to the vestibule could improve pain and sexual function in women with vestibulodynia and genitourinary syndrome of menopause [73]. Building on these findings, a randomized, double-blind, sham-controlled trial at three clinical sites evaluated the safety and efficacy of vestibule-targeted fractional CO₂ laser therapy for vestibular pain [74]. Seventy women were randomized in a 2:1 ratio to receive active treatment or sham procedures using the SmartXide2 V2LR MonaLisa Touch system. In the active arm, clinically meaningful reductions in vestibular pain were observed, accompanied by improvements in ICSI and ICPI scores (e.g., ICSI change from baseline to week 16: −0.91 to − 0.754; ICPI: −0.99). Additional benefits included better Vaginal Genital Tissue Assessment (VGTA) scores, reduced pain on cotton-swab testing, and improved sexual function and distress as measured by the FSFI and FSDS-R. Together, the pilot and randomized controlled data support fractional CO₂ laser therapy as a promising nonhormonal option for vulvovaginal pain.

### Integrating data science in BPS/IC and vulvodynia research

**Fig. 4:**
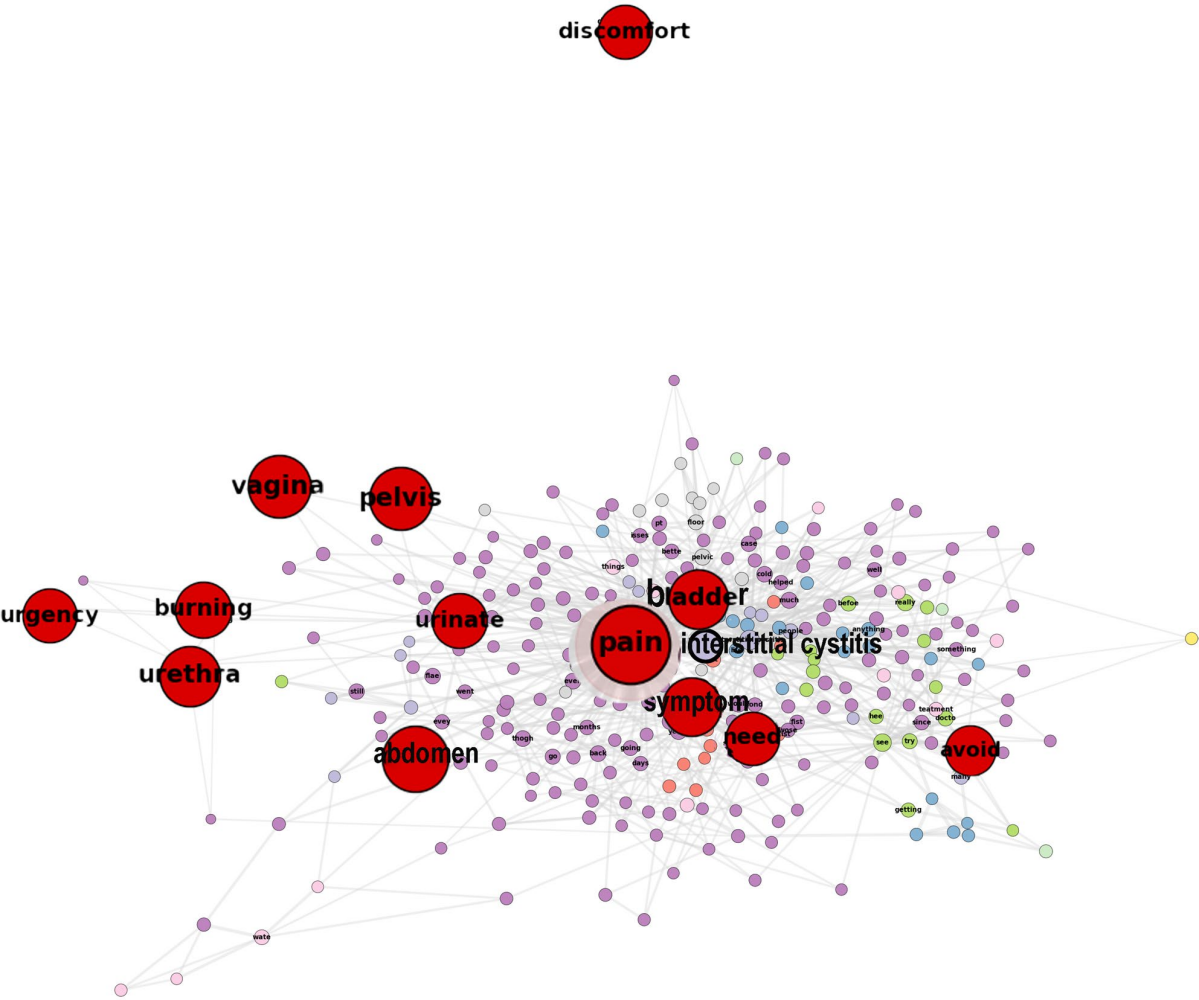
Community structure of BPS/IC -related vocabulary in social media discourse. Spring-layout visualization of the co-occurrence network, with ‘pain’ at the center. Red nodes indicate validated clinical terms from standardized questionnaires; other colored clusters represent thematic communities detected by the Louvain algorithm. Node size for clinical terms (red) has been exaggerated to emphasize key concepts relevant to BPS/IC and its comorbidities. The network shows a clear hub-and-spoke organization around clinical concepts with distinct peripheral clusters. Abbreviation: BPS/IC, interstitial cystitis/bladder pain syndrome

This highlights the need for dynamic and adaptive therapeutic approaches that accommodate individual symptom trajectories. An AI-based framework is currently under development to enable continuous re-clustering of patients and personalize therapeutic decisions accordingly [60]. Recent evidence further underscores that QoL stratification reveals distinct symptom patterns and comorbidities, including vulvodynia, within the BPS/IC population [76]. This highlights the need for dynamic and adaptive therapeutic approaches that accommodate individual symptom trajectories. An AI-based framework is currently under development to enable continuous re-clustering of patients and personalize therapeutic decisions accordingly [60]. Recent evidence further underscores that QoL stratification reveals distinct symptom patterns and comorbidities, including vulvodynia, within the BPS/IC population [76]. Natural language processing-based co-occurrence network analysis of pain-related expressions in social media has demonstrated that symptom-related vocabulary forms semantically distinct clusters and that key clinical descriptors such as burning are frequently used in metaphorical or aesthetic contexts (e.g., burning church, burning regret), indicating a semantic drift between clinical terminology and everyday digital discourse [77]. This lexical divergence may impair symptom recognition in younger or digitally native patients. Building on these insights, an AI-driven framework has been applied to BPS/IC -related clinical questionnaires and online discourse; Fig. 4 shows the resulting community structure of pain-related vocabulary in BPS/IC, with symptom expressions diverging into hierarchical clusters centered on core terms such as pain, headache, burning, and discomfort [77]. These structural insights may inform refinement of BPS/IC phenotypes and identification of clinically relevant symptom clusters [78], while mapping between clinical terminology and everyday digital discourse can facilitate the redesign of symptom instruments aligned with natural patient language [77].

### Future Perspectives

**Fig. 5:**
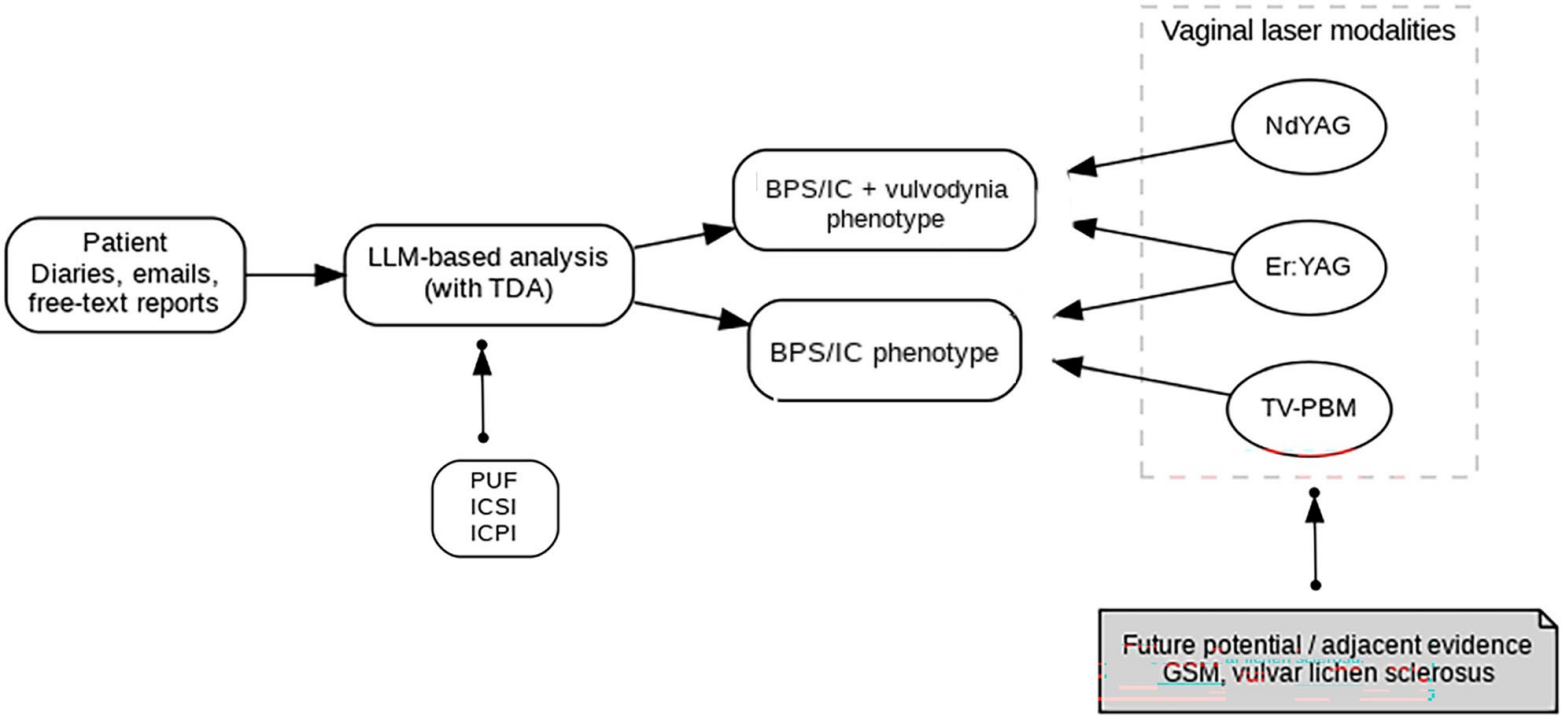
Conceptual framework for a future AI-driven learning healthcare system in BPS/IC and vulvodynia. Unstructured patient-generated data (diaries, emails, free-text reports) as inputs for LLM-based analysis (with Topological Data Analysis), informed by validated clinical scales (Pelvic Pain and Urgency/Frequency Patient Symptom Scale [PUF], Interstitial Cystitis Symptom Index [ICSI], Interstitial Cystitis Problem Index [ICPI]). AI-driven stratification into distinct phenotypes (e.g., BPS/IC-only vs. BPS/IC + vulvodynia) for personalized, mechanism-based treatment decisions, such as the selection of appropriate vaginal laser modalities. Future potential from adjacent evidence (GSM, vulvar lichen sclerosus) for emerging modalities (e.g., TV-PBM)

BPS/IC and vulvodynia are highly heterogeneous syndromes characterized by a complex interplay of pathophysiology, comorbidities, and psychosocial factors, making them intractable to one-size-fits-all treatment algorithms [1, 5, 15, 25, 58–61]. Recent international discussions have emphasized the importance of systematic evaluation of gynecological comorbidities, including vulvodynia and vestibulodynia, as a cornerstone of BPS/IC management, as well as the need for multifaceted clustering and precise phenotyping that incorporates health-related QoL, psychological characteristics, and pain distribution [1, 59, 79, 80]. As overviewed in this review, the high comorbidity rate of BPS/IC and vulvodynia, significant sexual dysfunction, the existence of nociplastic pain subgroups based on psychological and emotional traits [80], widespread pain distribution, and diverse resilience levels [81] strongly suggest a paradigm shift towards care based on a personalized medicine framework that designs “what to do, for whom, and in what order” [21, 22, 25, 59, 60, 79, 81, 82]. Three major challenges must be overcome to implement this personalization. First, stratification itself has not been sufficiently refined; multiaxial cluster analyses integrating clinical symptoms, gynecological findings, psychological traits, endocrine status, and QoL, as well as validation of treatment responses by phenotype, remain limited [1, 24, 58, 59, 79, 80, 82]. Second, standard operating procedures and safety profiles regarding energy settings, irradiation targets, and treatment schedules specific to each laser modality are not standardized, making comparisons between facilities and positioning relative to interventional studies (including other non-pharmacological therapies) difficult [9, 70, 83–87]. Third, outcome assessments remain skewed towards pain scores, and study designs incorporating multidimensional endpoints—such as bladder function indices, sexual function, psychological distress, activity levels, resilience, and quality of life—are insufficient [6, 7, 64, 80, 84, 85, 88–90]. Furthermore, current laser-related evidence is centered on case series and non-controlled studies; their methodological limitations and heterogeneity have been clearly noted in recent reviews and meta-analyses covering interventions and flares in BPS/IC and Urologic Chronic Pelvic Pain Syndrome (UCPPS) [9, 84–86, 90, 91]. To overcome these challenges and move closer to true personalized implementation, it is also crucial to bridge the lexical gap between clinical terminology and the natural language patients use daily [77]. The vocabulary used to describe symptoms is subject to “semantic drift,” where meanings diverge between the clinical setting and the digital space, potentially affecting symptom recognition and the validity of questionnaires and Patient-Reported Outcome (PRO) measures [77, 92, 93]. For redesigning next-generation stratification and assessment tools, this paper proposes three pillars: (1) continuous collection of patient diaries, free-text descriptions, and electronic PROs; (2) time-series analysis of lexical networks and discourse context using Large Language Models (LLMs); and (3) bidirectional mapping between clinical terminology and natural language vocabulary [24, 63, 75–77]. This approach is expected to enable the construction of dynamic, subtype-specific assessment tools aligned with the words patients actually use, contributing to questionnaire updates, optimization of educational materials, and reduction of diagnostic delays, especially among the digital-native generation [24, 93, 75–77, 80, 92, 93]. Figure 5 illustrates the conceptual framework of such a Learning Health System. It visualizes a process where AI-driven analysis of unstructured patient-generated data (e.g., diaries, free-text reports) is augmented by validated clinical indices (like PUF, ICSI, ICPI) and existing BPS/IC and Urologic Chronic Pelvic Pain Syndrome specific scales [80, 84, 85, 90]. This stratification of patients into different phenotypes, such as BPS/IC-only versus BPS/IC+vulvodynia, connects to mechanism-based treatment decisions, including the selection of specific vaginal laser modalities or other non-pharmacological interventions [79–81, 83, 84, 86, 88–90, 93, 94].

Redefining laser therapy within this framework, its value lies in integrating modality-specific biological actions “for the right purpose, in the right subtype.” Non-ablative Er:YAG lasers may induce mucosal and supporting tissue repair via heat shock responses and microstructural remodeling. Conversely, Nd:YAG lasers, with their deeper tissue penetration and hemoglobin selectivity, may contribute to the physiological modulation of soft tissues from the vulva to the pelvic floor. For Hunner’s lesions, local treatments such as endoscopic ablation or transurethral coagulation remain practical options [94]. In clusters with minimal inflammatory findings where pelvic myalgia is predominant, validating the analgesic and anti-inflammatory effects of TV-PBM in combination with non-pharmacological therapies and exercise therapy is warranted [83, 84, 88, 96, 97]. Through such mechanism-based modularization, the therapeutic role of lasers (first-line, adjunctive, salvage) can be strategically assigned by subtype. Furthermore, in subgroups with menopause-related changes or androgen deficiency, a rational strategy combining laser therapy with hormone or androgen therapy can be envisioned to improve sexual function and the mucosal environment. Findings on laser therapy in adjacent disease areas, such as GSM and vulvar lichen sclerosus, should not be considered direct evidence for BPS/IC or vulvodynia. Instead, they should be positioned as mechanistic support regarding effects on epithelial, vascular, and neural modulation within the same anatomical region—i.e., as “future possibilities/related evidence”—and are thus placed in the “adjacent evidence” domain of Fig. 5 [84, 87, 88, 91]. Future priorities include implementing adaptive or stratified randomized controlled trials that incorporate pre-treatment phenotyping [79, 85, 90, 97]; standardizing irradiation conditions and exposure metrics for each laser modality; adopting assessment frameworks that emphasize multidimensional endpoints and PROs [80, 84, 85, 88–90]; validating combination strategies with hormone therapy, pelvic floor rehabilitation, psychotherapy/cognitive-behavioral therapy, exercise therapy, acupuncture, and biofeedback [83, 84, 86, 87, 89, 91, 93, 96–99]; and introducing a Learning Health System that integrates LLM-based analysis of patient diaries and lexical mapping into real-time clinical decision-making [79, 80, 85, 90, 92, 93]. As outlined in this review, laser therapy may demonstrate its true value in pelvic pain syndromes with overlapping BPS/IC and vulvodynia when incorporated into personalized strategies as a mechanism-specific module [83, 84, 86, 87, 89, 91, 95–99]. Within such a framework, improved outcomes and quality of life for these complex, overlapping pelvic pain syndromes are anticipated through iterative treat-to-target optimization [79, 80, 82, 84, 85, 88–91, 93, 98].

## Conclusions

Bladder pain syndrome/interstitial cystitis (BPS/IC) and vulvodynia represent complex, overlapping pelvic pain syndromes requiring individualized, mechanism-based, and multidisciplinary management. As summarized in this review, current evidence—though limited and heterogeneous—supports the biological plausibility of vaginal and vulvar laser therapies as targeted modules within personalized care frameworks. Moving forward, clinical progress will depend on standardized protocols, stratified and adaptive randomized trials, multidimensional patient-reported endpoints, and integration of AI-assisted phenotyping into daily practice. By situating laser therapy as one component within a broader personalized medicine strategy, improved symptom control, sexual function, and quality of life can be expected for women affected by these challenging comorbid pain syndromes.

## Data Availability

No datasets were generated or analysed during the current study.

## Abbreviations

BPS/IC: Bladder pain syndrome/interstitial cystitis
Er:YAG: Erbium-doped yttrium aluminum garnet
Nd:YAG: Neodymium-doped yttrium aluminum garnet
Ho:YAG: Holmium-doped yttrium aluminum garnet
FSFI: Female Sexual Function Index
FSDS-R: Female Sexual Distress Scale-Revised
QoL: Quality of life
TV-PBM: Transvaginal photobiomodulation
